# Comparative Efficacy and Safety of Poly (ADP-Ribose) Polymerase Inhibitors in Patients With Ovarian Cancer: A Systematic Review and Network Meta-Analysis

**DOI:** 10.3389/fonc.2022.815265

**Published:** 2022-06-08

**Authors:** Jing Luo, Shunlong Ou, Hua Wei, Xiaoli Qin, Qian Jiang

**Affiliations:** ^1^ School of Medicine, University of Electronic Science and Technology of China, Chengdu, China; ^2^ Sichuan Cancer Hospital & Institute, Sichuan Cancer Centre, School of Medicine, University of Electronic Science and Technology of China, Chengdu, China; ^3^ Department of Pharmacy, Dujiangyan People’s Hospital, Dujiangyan Medical Center, Dujiangyan, China; ^4^ School of Pharmacy, Chengdu Medical College, Chengdu, China

**Keywords:** PARP inhibitors, ovarian cancer, olaparib, niraparib, rucaparib, network meta-analysis

## Abstract

**Objective:**

This study aims to compare the efficacy and safety of different poly (ADP-ribose) polymerase (PARP) inhibitors in patients with ovarian cancer through a network meta-analysis to support clinical treatment choices.

**Methods:**

The Cochrane Library, PubMed, Embase, Science Citation Index, China National Knowledge Infrastructure (CNKI), Wanfang Data, Chongqing VIP (CQVIP), and Chinese BioMedical Literature Database (CBM) were searched with a cutoff date of 14 January 2021. ClinicalTrials.gov was also checked for supplementary data. Phase II or III randomized controlled trials that compared a PARP inhibitor with a placebo in patients with relapsed or newly diagnosed advanced ovarian cancer were included. The hazard ratios (HRs) for progression-free survival and overall survival and odds ratios (ORs) for grade 3 or higher adverse events were analyzed. The network meta-analysis was conducted in a Bayesian framework based on the Markov Chain Monte Carlo model in the R gemtc package (version 4.0.3).

**Results:**

Eight eligible articles reporting six trials with a total of 2,801 patients were incorporated in this network meta-analysis. Three trials compared olaparib with placebo. Two trials compared niraparib with placebo. One trial compared rucaparib with placebo. The network meta-analysis failed to show significant differences in progression-free survival among the three PARP inhibitors: HR of 0.64, 95% confidence interval of 0.3 to 1.42 for olaparib versus niraparib, and olaparib versus rucaparib (0.86; 0.33 to 2.33). The comparison between niraparib and rucaparib also did not express a statistical difference (1.34; 0.47 to 3.72). Subgroup analysis bybreast cancer susceptibility gene (BRCA) status showed no obvious difference in progression-free survival among the three PARP inhibitors regardless of BRCA mutation status. Olaparib had fewer grade 3 or higher adverse events than niraparib (OR, 0.27; 95% confidence interval, 0.13 to 0.55) and rucaparib (0.34; 0.14 to 0.86). However, the analysis failed to show a significant difference between niraparib and rucaparib (1.27; 0.49 to 3.27).

**Conclusion:**

Current evidence indicates that there is no significant difference observed in efficacy among olaparib, niraparib, and rucaparib. However, olaparib might have fewer grade 3 or higher adverse events.

## Introduction

Ovarian cancer is the eighth leading cause of cancer death in women, with an estimated 210,000 deaths globally in 2020, making it the second leading cause of gynecologic cancer death (1). It is worth noting that there is still a lack of typical clinical manifestations and early diagnosis methods, and more than 70% of patients are diagnosed with an advanced stage (2). To date, surgery, systemic therapy, targeted therapy, and radiotherapy have remained the mainstays of therapy for ovarian cancer. Unfortunately, most patients will recur or progress after completing platinum-based chemotherapy. Moreover, in countries with abundant medical resources, compared with the 85% 5-year survival rate for breast cancer, ovarian cancer after diagnosis is only 47% (3).

A deeper understanding of the molecular mechanisms of tumors has promoted an influx of innovative therapies, particularly targeted drugs and immunotherapies, which have marked major therapeutic advances in oncology (4, 5). Poly (ADP-ribose) polymerase (PARP) is a DNA repair enzyme that can recognize and bind to DNA breaks to activate and catalyze the poly-ADP ribosylation of receptor proteins and participate in the DNA repair process. PARP inhibitors play a critical role in blocking DNA single-strand break repair, resulting in the accumulation of double-strand breaks, which cannot be entirely repaired in tumors with homologous recombination deficiency (HRD), especially in ovarian cancer patients with breast cancer susceptibility gene (BRCA)1/BRCA2 mutations. It can lead to cell death through the process of synthetic lethality caused by mutation or loss of function genes (6–8), providing an example of the precision medicine in recent decades (9, 10).

Currently, olaparib, niraparib, rucaparib, and talazoparib have acquired regulatory approval from the US Food and Drug Administration (FDA) for use in ovarian cancer, breast cancer, etc. At the same time, a series of evidence showed that PARP inhibitors as maintenance therapy for ovarian cancer could significantly delay the time to recurrence and extend progression-free survival (PFS) (11–14). Interestingly, PARP inhibitors were more likely to improve PFS in ovarian cancer patients with or without BRCA mutations and HRD (15).

Network meta-analysis can be used for indirect comparison of multiple treatment options, allowing multiple interventions to incorporate direct and indirect comparisons (16). Previous studies have confirmed the efficacy of PARP inhibitors in patients with ovarian cancer while considering that some research results have been updated. Consequently, we conducted this study to further investigate the efficacy and safety of different PARP inhibitors in ovarian cancer treatment and to provide the best clinical choice.

## Methods and Analysis

Our study followed the Preferred Reporting Items for Systematic Reviews and Meta-Analyses (PRISMA) extension statement for network meta-analysis (**Supplementary Table S1**). The study was registered on the International Prospective Register of Systematic Reviews (PROSPERO, CRD4202020214).

### Data Sources and Search Strategy

We systematically searched eight databases, including Cochrane Library, PubMed, Embase, Science Citation Index, China National Knowledge Infrastructure (CNKI), Wanfang Data, Chongqing VIP (CQVIP), and Chinese BioMedical Literature Database (CBM) with a cutoff date of 14 January 2021. The retrieval strategy is detailed in **Supplementary Table S2**. Both MeSH and free text terms were used to identify relevant articles. Furthermore, reference lists of pertinent retrieved articles were reviewed for additional studies, and ClinicalTrials.gov was also checked to ensure that data from previously published trials were updated on the registry.

### Inclusion and Exclusion Criteria

Phase II or III randomized controlled trials (RCTs) were included if they met the following criteria: (1) adult women with ovarian cancer; (2) the intervention was one of the following PARP inhibitors, including olaparib, niraparib, rucaparib, veliparib, fluzoparib, talazoparib, and pamiparib; (3) the comparison was treated with a placebo; and (4) outcome: trials reported at least one of the following outcome measures: PFS, overall survival (OS), and grade 3 or higher adverse events (AEs).

We excluded studies based on the following criteria: (1) duplicate publication or conference abstract, (2) studies with outdated preliminary results, and (3) trials with unavailable data.

### Data Extraction

All data were collected using structured Excel sheets, including but not limited to the trial name, the first author, publication or presentation year, trial design, phase, cancer type, number of patients, type of PARP inhibitors, use of control group, dosing schedule, follow-up time, reported outcomes, and methodological information for all eligible studies. Two reviewers independently extracted the data. Any disagreement was resolved by discussion or consultation with a third reviewer.

### Risk-of-Bias Assessment

The assessment was based on the Cochrane risk-of-bias tool (17) using Review Manager 5.3 software, including selection bias (random sequence generation and allocation concealment), performance bias (blinding of participants and personnel), detection bias (blinding of outcome assessment), attrition bias (incomplete outcome data), reporting bias (selective reporting), and other potential sources of bias. The risk-of-bias assessment was classified as low risk, unclear, and high risk.

### Data Synthesis and Statistical Analysis

We used hazard ratios (HRs) for survival outcomes (PFS and OS) and odds ratios (ORs) for binary outcomes (grade 3 and higher AEs), providing a 95% confidence interval (CI) as a measure of effect for analysis. The pooled HR with 95% CI was calculated by inverse variance.

Network plots were generated based on different outcomes by using Stata 15.0 software. All network meta-analyses were conducted in the Bayesian framework based on the Markov Chain Monte Carlo model in the R gemtc package (version 4.0.3). For outcomes, each model used four Markov chains for initial value setting with 5,000 burn-ins, 20,000 iterative operations, and a thinning interval of 1. The convergence of the model was evaluated through the Brooks–Gelman–Rubin diagnosis plot, trace plot, and density plot. Simultaneously, the deviance information criterion (DIC) was used to assess the fit of the consistency model and inconsistency model.

Meanwhile, considering the heterogeneity of the included studies, we adopted the random-effects model and used *I* ^2^ statistics in the visual forest plot to evaluate the heterogeneity between the studies. Subgroup analysis was conducted based on BRCA status. Sensitivity analysis was performed to assess the stability of the results by restricting the drug dose.

## Results

### Systematic Review and Characteristic

**Figure 1:**
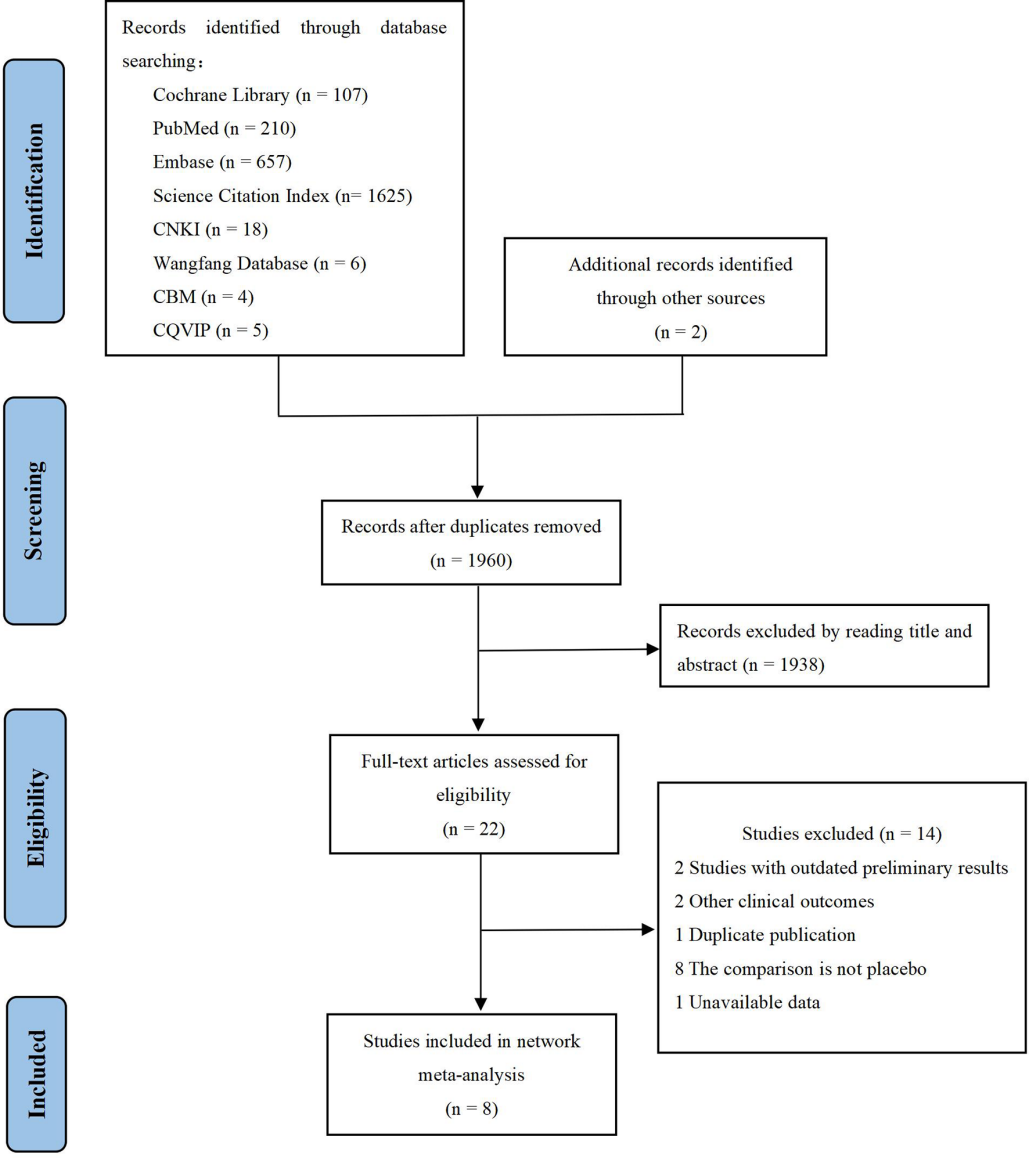
Flow diagram of study selection.

**Figure 2:**
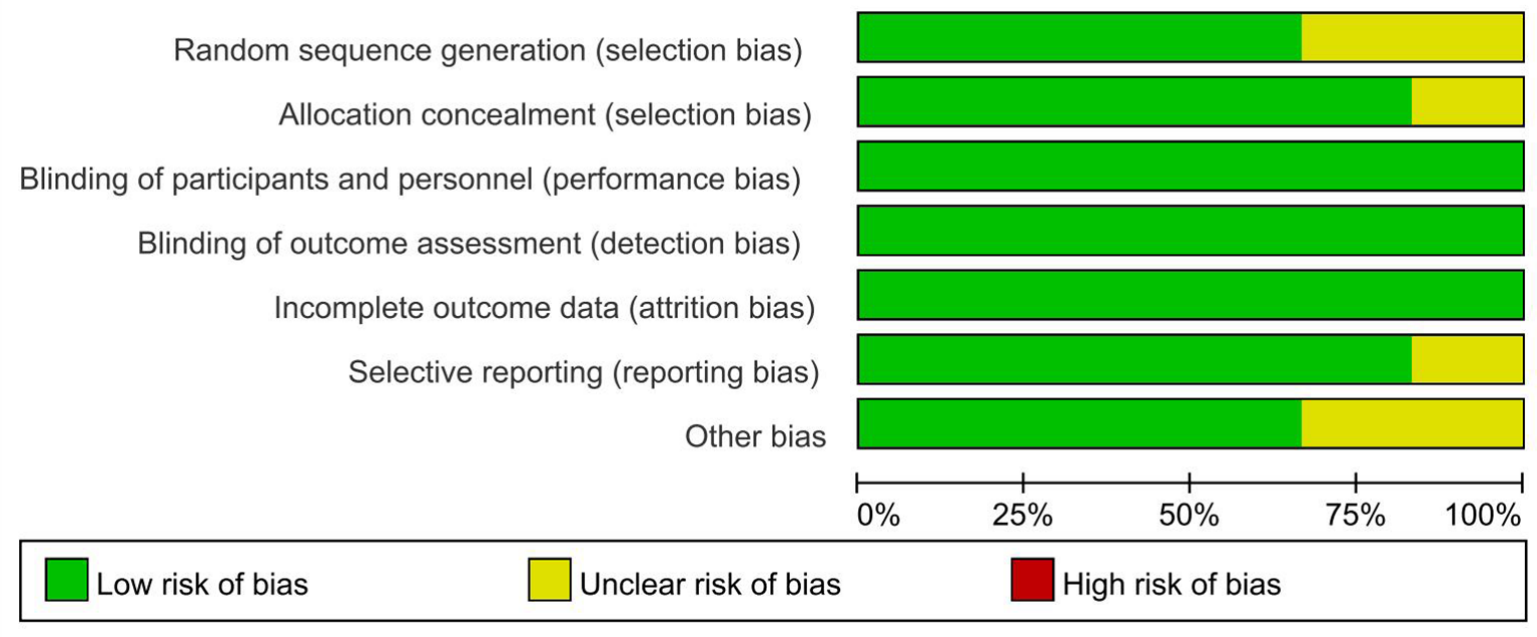
Risk of bias graph.

We identified 2,634 records through initial retrieval. After duplication and abstract screening, 22 records were reviewed in full text. Ultimately, eight articles (11–13, 18–22) reporting six RCTs were considered eligible for this network meta-analysis with a total of 2,801 patients (**Figure 1**), of which three trials compared olaparib with placebo, two trials compared niraparib with placebo, and one trial compared rucaparib with placebo (**Table 1**). In total, four trials targeted the treatment of patients with platinum-sensitive recurrent ovarian cancer and two trials for newly diagnosed advanced ovarian cancer. Notably, two articles in Study 19 were eligible for analysis, with Ledermann et al. (21) reporting the results of PFS, OS, and grade ≥3 AEs (58% maturity), and Friedlander et al. (18) reporting updated OS (79% maturity). For ARIEL3, Ledermann et al. (22) only reported the updated results of grade ≥3 AEs, while the results of PFS were still derived from the paper in 2017 (13). The risk-of-bias assessments are summarized in **Figure 2**.

**Table 1:** Baseline characteristics of included studies.

First author	Year	Registry number	Study code	Phase	Setting	Sample (Int/Con)	Intervention arm	Control arm	BRCA status		Follow-up (month)	Reported outcomes
									No. of BRCAm patients (Int/Con)	No. of BRCAw patients (Int/Con)		
Ledermann (18, 21)	2014	NCT00753545	Study 19	II	Platinum-sensitive relapsed ovarian cancer	265 (136/129)	Olaparib 400 mg twice daily	Placebo	136^a^ (74/62)	118 (57/61)	78^*^	PFS, OS, grade ≥3 AEs
Pujade-Lauraine (12)	2017	NCT01874353	SOLO2	III	Platinum-sensitive relapsed ovarian cancer	295 (196/99)	Olaparib 300 mg twice daily	Placebo	295^b^ (196/99)	0	22	PFS, OS, grade ≥3 AEs
Moore (19)	2018	NCT01844986	SOLO1	III	Newly diagnosed advanced ovarian cancer	391 (260/131)	Olaparib 300 mg twice daily	Placebo	391^a^ (260/131)	0	41	PFS, OS, grade ≥3 AEs
Mirza (11)	2016	NCT01847274	NOVA	III	Platinum-sensitive relapsed ovarian cancer	553 (372/181)	Niraparib 300 mg once daily	Placebo	203^b^ (138/65)	249 (163/86)	16.9	PFS, grade ≥3 AEs
González-Martín (20)	2019	NCT02655016	PRIMA	III	Newly diagnosed advanced ovarian cancer	733 (487/246)	Niraparib 300 mg once daily	Placebo	223^a^ (152/71)	399 (264/135)	13.8	PFS, OS, grade ≥3 AEs
Coleman (13, 22)	2017	NCT01968213	ARIEL3	III	Platinum-sensitive relapsed ovarian cancer	564 (375/189)	Rucaparib 600 mg twice daily	Placebo	196^a^ (130/66)	368 (245/123)	36	PFS, grade ≥3 AEs

Int, intervention arm; Con, control arm; No., number; BRCAm, BRCA mutated; BRCAw, BRCA wild-type; PFS, progression-free survival; OS, overall survival; AEs, adverse events.

^*^Study 19 corresponded to 79% OS data maturity with a median follow-up of 78.0 months.

a Patients with a germline or somatic BRCA mutation.

b Patients with a germline BRCA mutation.

### Network Meta-Analysis

**Figure 3:**
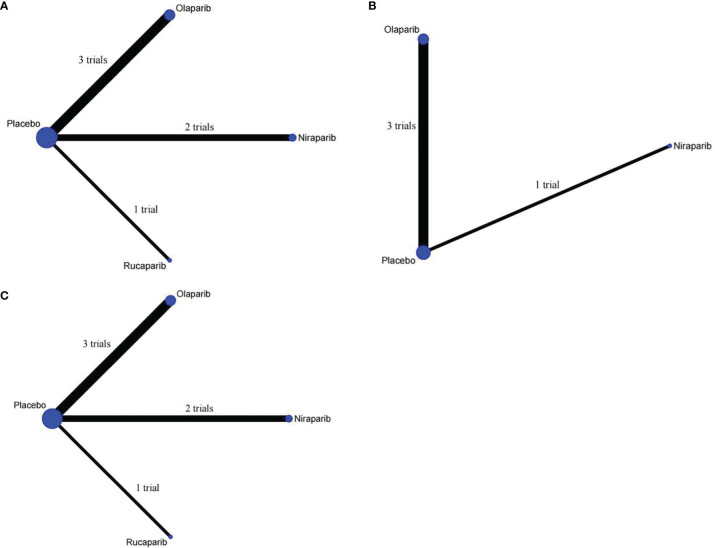
Network plot of different outcomes in patients with ovarian cancer. **(A)** Comparisons on progression-free survival in patients with ovarian cancer. **(B)** Comparisons on overall survival in patients with ovarian cancer. **(C)** Comparisons on grade ≥3 adverse events in patients with ovarian cancer.

**Figure 4:**
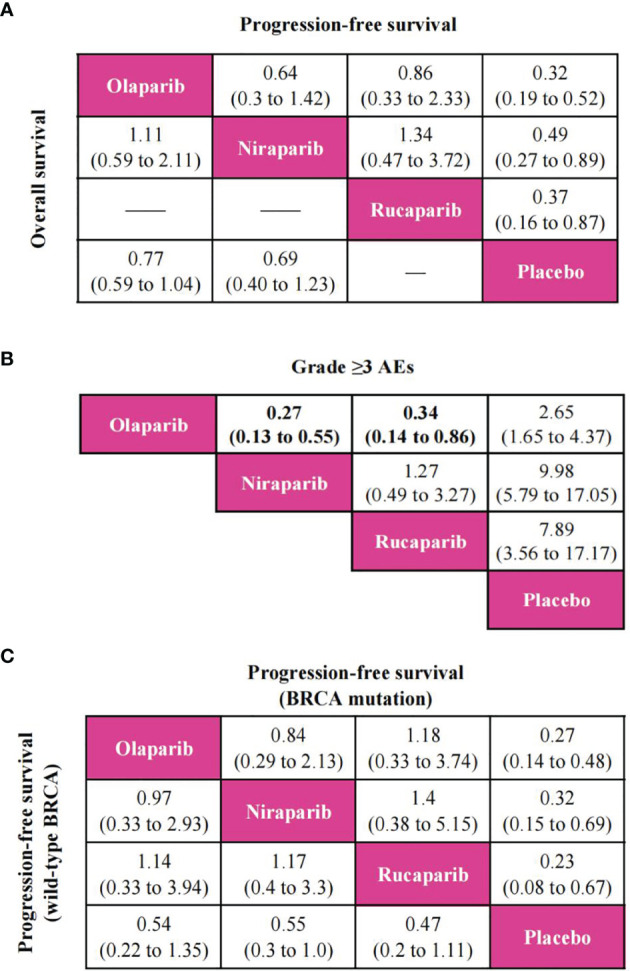
Pooled estimates of the network meta-analysis. **(A)** Pooled hazard ratios (95% confidence intervals) for progression-free survival (upper triangle) and overall survival (lower triangle) in the overall population. **(B)** Pooled odds ratios (95% confidence intervals) for grade ≥3 adverse events in the overall population. **(C)** Pooled hazard ratios (95% confidence intervals) for progression-free survival of BRCA mutation population (upper triangle) and wild-type BRCA population (lower triangle).

PFS was reported in all trials, and the network plot was presented in **Figure 3A**. In terms of PFS (**Figure 4A**), compared with placebo, three PARP inhibitors showed significant benefits (olaparib (HR, 0.32; 95% CI, 0.19 to 0.52), niraparib (0.49; 0.27 to 0.89), and rucaparib (0.37; 0.16 to 0.87)). Nevertheless, the network meta-analysis failed to show significant differences in progression-free survival among the three PARP inhibitors: 0.64; 0.3 to 1.42 for olaparib versus niraparib and 0.86; 0.33 to 2.33 for olaparib versus rucaparib. The comparison between niraparib and rucaparib also did not express a statistical difference (1.34; 0.47 to 3.72).

In terms of OS, since ARIEL3 did not report OS, only olaparib and niraparib were compared in this analysis (**Figure 3B**). Notably, the results failed to show statistical differences between the two PARP inhibitors and placebo [olaparib versus placebo (0.77; 0.59 to 1.04), niraparib versus placebo (0.69; 0.40 to 1.23)]. Moreover, there was no obvious difference between olaparib and niraparib [olaparib versus niraparib (1.11, 0.59 to 2.11)], as shown in **Figure 4A**.

Network meta-analysis included all trials for Grade ≥3 AEs (**Figure 3C**). The results of grade ≥3 AEs are shown in **Figure 4B**; compared with placebo, the three PARP inhibitors had more toxicity [olaparib (OR, 2.65; 95% CI, 1.65 to 4.37), niraparib (9.98; 5.79 to 17.05), and rucaparib (7.89; 3.56 to 17.17)]. Olaparib had fewer grade ≥3 AEs among the three PARP inhibitors [versus niraparib (0.27; 0.13 to 0.55), rucaparib (0.34; 0.14 to 0.86)]. However, we observed no significant difference with niraparib versus rucaparib (1.27; 0.49 to 3.27).

### Subgroup Analysis

According to available data in patients with BRCA mutation or wild-type BRCA, we only performed a PFS network meta-analysis, which included six trials for the BRCA mutation population and four trials for the wild-type BRCA population (**Supplementary Figure S3**). As shown in **Figure 4C**, three PARP inhibitors were associated with the benefits of PFS compared to placebo in patients with BRCA mutation. Nevertheless, there was no statistically significant difference in progression-free survival among the three PARP inhibitors regardless of BRCA mutation status.

### Heterogeneity and Inconsistency Assessment

The fit of the consistency model was similar to the inconsistency model (**Supplementary Table S3**). We observed low heterogeneity (*I* ^2^ = 0%) in most comparisons of overall and subgroup populations. Moreover, moderate to high heterogeneity was discovered in the following comparisons: (1) niraparib versus placebo for PFS (*I* ^2^ = 87.5%) in the overall population and (2) niraparib versus placebo (*I* ^2^ = 42.8%) and olaparib versus placebo (*I* ^2 =^ 40.9%) for PFS in the BRCA mutation subgroup (**Supplementary Figure S2**).

### Sensitivity Analysis

We observed that olaparib was used in different doses with 300 or 400 mg twice daily. Consequently, we unified the dose to 300 mg twice daily (excluding Study 19) in the sensitivity analysis with a total of 2,536 patients. When comparing the original network meta-analysis, the results were consistent without any deviation (**Supplementary Figure S4**, **S5**), indicating that the results of this study were robust.

## Discussion

### Main Findings

This study explored the diversity of efficacy and safety of different PARP inhibitors in patients with ovarian cancer through a network meta-analysis. Firstly, the results of a network meta-analysis based on available evidence showed that the three PARP inhibitors (olaparib, niraparib, and rucaparib) could prolong PFS over a placebo. It is probably worth noting that their long-term benefit was not limited to BRCA mutation status. Nevertheless, no significant differences were discovered among the three PARP inhibitors in patients with ovarian cancer. Meanwhile, the analysis indicated that there was no difference in OS between two PARP inhibitors (olaparib and niraparib) and placebo. Secondly, the results implied that olaparib had the fewest grade 3 or higher adverse events, and no difference was observed between niraparib and rucaparib.

### Strengths and Implications

To date, there has never been a randomized controlled trial that directly compares different PARP inhibitors. This study used network meta-analysis to compare the efficacy and safety of PARP inhibitors in patients with relapsed or newly diagnosed advanced ovarian cancer, providing an example of a systematic methodology to generate reliable evidence. In this context, this network meta-analysis based on indirect evidence can support clinical treatment choices. A Bayesian method was used to conduct this network meta-analysis, and the uncertainty of the model was fully considered (23). Moreover, we considered that different doses of the same drug may compromise the consistency and quality of the results. Therefore, we performed a sensitivity analysis using the drug dose as a qualifier (24), and the results also confirmed the stability of the overall results, making them reliable.

Targeted therapies based on PARP inhibitors are essential for the maintenance treatment of first-line or relapsed ovarian cancer patients (25). A series of clinical trials using PARP inhibitors showed that the response of PARP inhibitors was strongly associated with platinum sensitivity (26). Compared with patients with non-BRCA-associated ovarian cancer, patients with BRCA germline mutation were more likely to have increased platinum sensitivity, improved survival, and high sensitivity to PARP inhibitors (27). Consequently, the importance of confirming cancer settings, the timing of treatment, and biomarkers was increasingly reflected in clinical practice.

There were hematologic adverse reactions to different PARP inhibitors, and the most common adverse event was anemia. The key mechanism of anemia caused by PARP inhibitors might be due to the loss of PARP-2, which affects the differentiation of erythroid progenitors (28). Niraparib was more likely to cause the occurrence of grade ≥3 AEs than olaparib. Compared with other PARP inhibitors, niraparib was associated with a higher risk of thrombocytopenia and neutropenia (29). It is worth noting that adverse events could be reduced by dose adjustment. The higher initial dose of niraparib might be associated with adverse events, and an initial dose of 200 mg daily was recommended for patients with a body weight of <77 kg or a platelet count of <150 × 10^9^/L (30).

### Limitations

Nevertheless, there are several limitations to our study. Firstly, in the absence of direct evidence for comparison, comparisons of different PARP inhibitors were based entirely on indirect evidence, leading to results that depended on the transitivity and consistency of included studies. Furthermore, we considered methodological heterogeneity; a random-effects model was used for data consolidation analysis. Secondly, because of the small number of included studies, we did not assess publication bias by using funnel plots, which might affect the accuracy of the results (31). Thirdly, there were differences in the follow-up time of included studies and low data maturity. For example, rucaparib did not report OS, and the results for OS needed to be updated later. Therefore, PFS was taken as the primary outcome in this study.

## Conclusion

In conclusion, the results support the use of PARP inhibitors for ovarian cancer, but there is no statistically significant difference observed in efficacy among olaparib, niraparib, and rucaparib. Moreover, olaparib might have fewer grade 3 or higher adverse events. Clinicians should consider the management of adverse events associated with PARP inhibitors in clinical practice.

## Data Availability Statement

The original contributions presented in the study are included in the article/**Supplementary Material**. Further inquiries can be directed to the corresponding author.

## Author Contributions

JL: data analysis and interpretation, drafting the manuscript, final approval of the version to be published, and study conception and design. SO: data analysis and revising it for important intellectual content. HW: data analysis and interpretation. XQ: data acquisition and double-checking. QJ: study conception and design and final approval of the version to be published. All authors listed have made a substantial, direct, and intellectual contribution to the work and approved it for publication.

## Funding

This study has received funding from the Beijing Medical and Health Foundation (B20021CS).

## Conflict of Interest

The authors declare that the research was conducted in the absence of any commercial or financial relationships that could be construed as a potential conflict of interest.

## Publisher’s Note

All claims expressed in this article are solely those of the authors and do not necessarily represent those of their affiliated organizations, or those of the publisher, the editors and the reviewers. Any product that may be evaluated in this article, or claim that may be made by its manufacturer, is not guaranteed or endorsed by the publisher.

## Supplementary Material

Supplementary Table 1 Checklist of the PRISMA extension for network meta-analysis.

Supplementary Table 2 Literature retrieval strategy.

Supplementary Table 3 Comparisons of the fit of consistency and inconsistency models.

Supplementary Figure 1 Convergence of the four chains established by inspection of trace plot, density plot, and Brooks-Gelman-Rubin diagnosis plot for progression-free survival **(A, B)**, overall survival **(C, D)**, and grade ≥3 adverse events **(E, F)**.

Supplementary Figure 2 Forest plot of heterogeneity in comparisons of overall and subgroup population for different outcomes. **(A)** Progression-free survival of overall population. **(B)** Overall survival of overall population. **(C)** Grade ≥3 adverse events of overall population. **(D)** Progression-free survival of BRCA mutation population. **(E)** Progression-free survival of wild-type BRCA population.

Supplementary Figure 3 Network plot of comparisons on progression-free survival of treatments in subgroups of patients with ovarian cancer. **(A)** Comparisons on progression-fee survival in BRCA mutation population. **(B)** Comparisons on progression-fee survival in wild-type BRCA population.

Supplementary Figure 4 Network plot of different outcomes in patients with ovarian cancer for sensitivity analysis. **(A)** Comparisons on progression-free survival in patients with ovarian cancer. **(B)** Comparisons on overall survival in patients with ovarian cancer. **(C)** Comparisons on grade ≥3 adverse events in patients with ovarian cancer.

Supplementary Figure 5 Pooled estimates of the sensitivity analysis. **(A)** Pooled hazard ratios (95% confidence intervals) for progression-free survival (upper triangle) and overall survival (lower triangle). **(B)** Pooled odds ratios (95% confidence intervals) for grade≥3 adverse events.
