# Supplementary figures and images for: Comparative Efficacy and Safety of Poly (ADP-Ribose) Polymerase Inhibitors in Patients With Ovarian Cancer: A Systematic Review and Network Meta-Analysis
Source: Front Oncol. 2022 Jun 8;12:815265. doi: 10.3389/fonc.2022.815265 (PMC9213680; doi:10.3389/fonc.2022.815265)

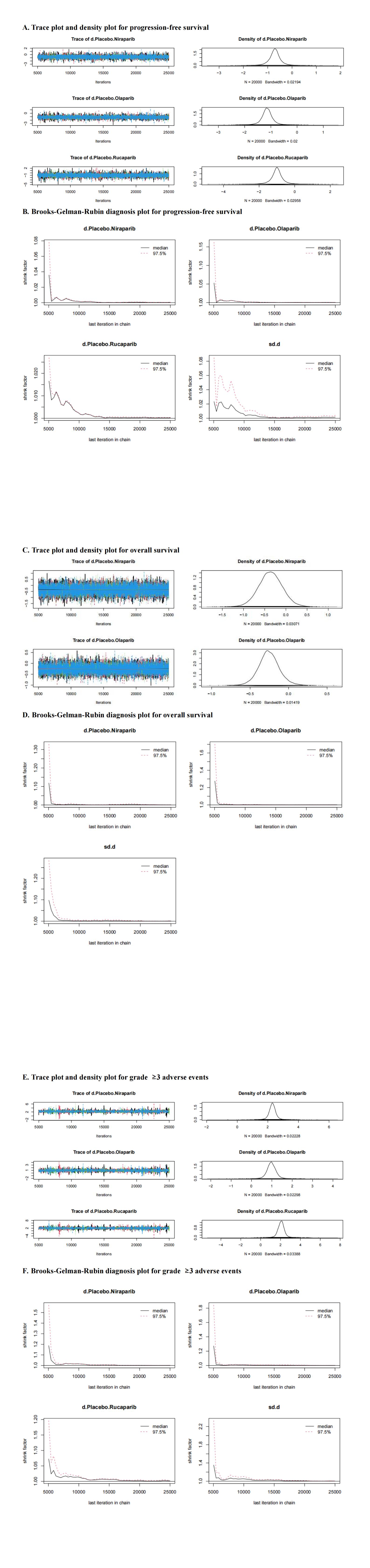

Supplement: Supplementary Figure 1 — Convergence of the four chains established by inspection of trace plot, density plot, and Brooks-Gelman-Rubin diagnosis plot for progression-free survival (A, B), overall survival (C, D), and grade ≥3 adverse events (E, F). [file Image_1.jpeg]

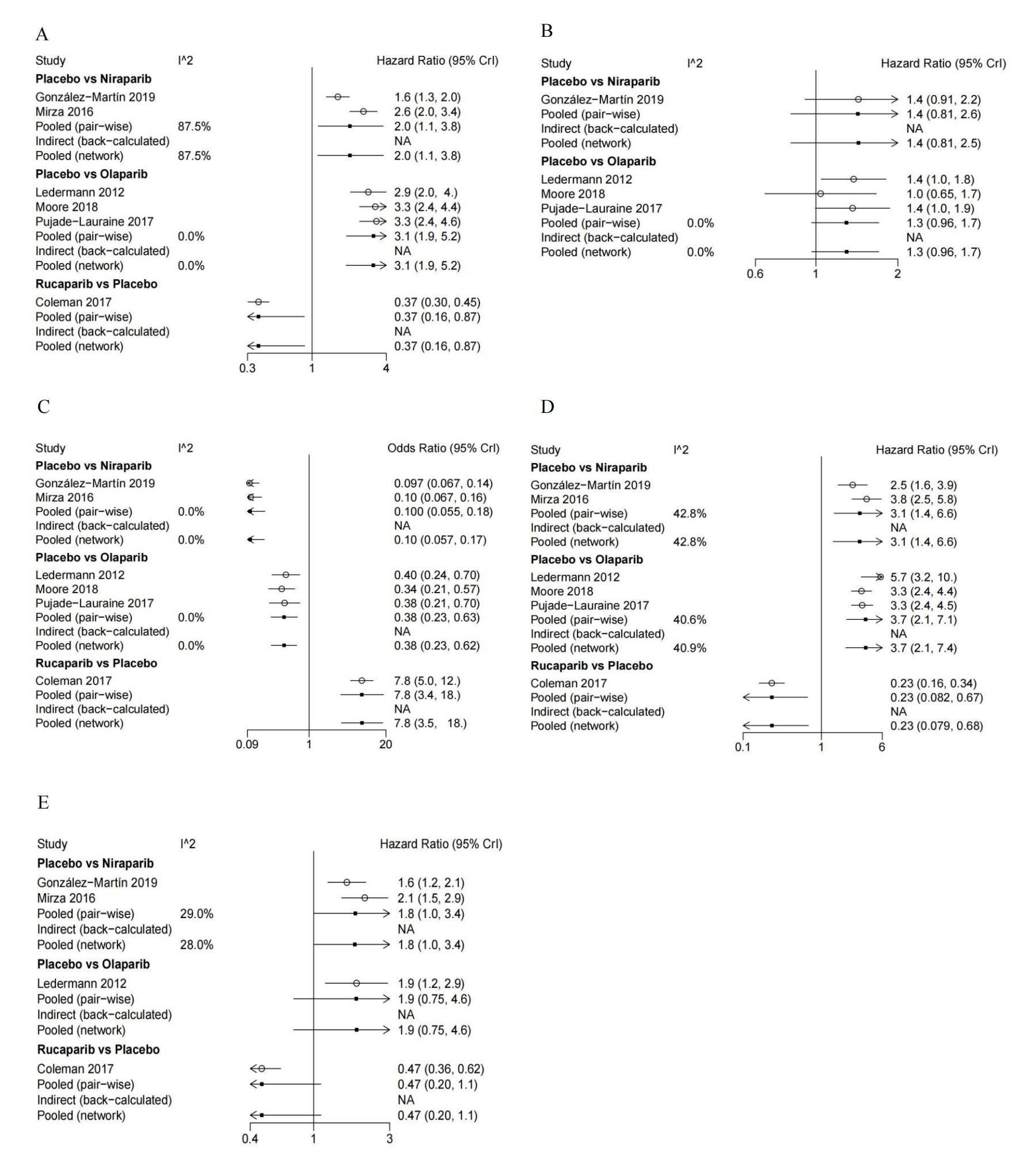

Supplement: Supplementary Figure 2 — Forest plot of heterogeneity in comparisons of overall and subgroup population for different outcomes. (A) Progression-free survival of overall population. (B) Overall survival of overall population. (C) Grade ≥3 adverse events of overall population. (D) Progression-free survival of BRCA mutation population. (E) Progression-free survival of wild-type BRCA population. [file Image_2.jpeg]

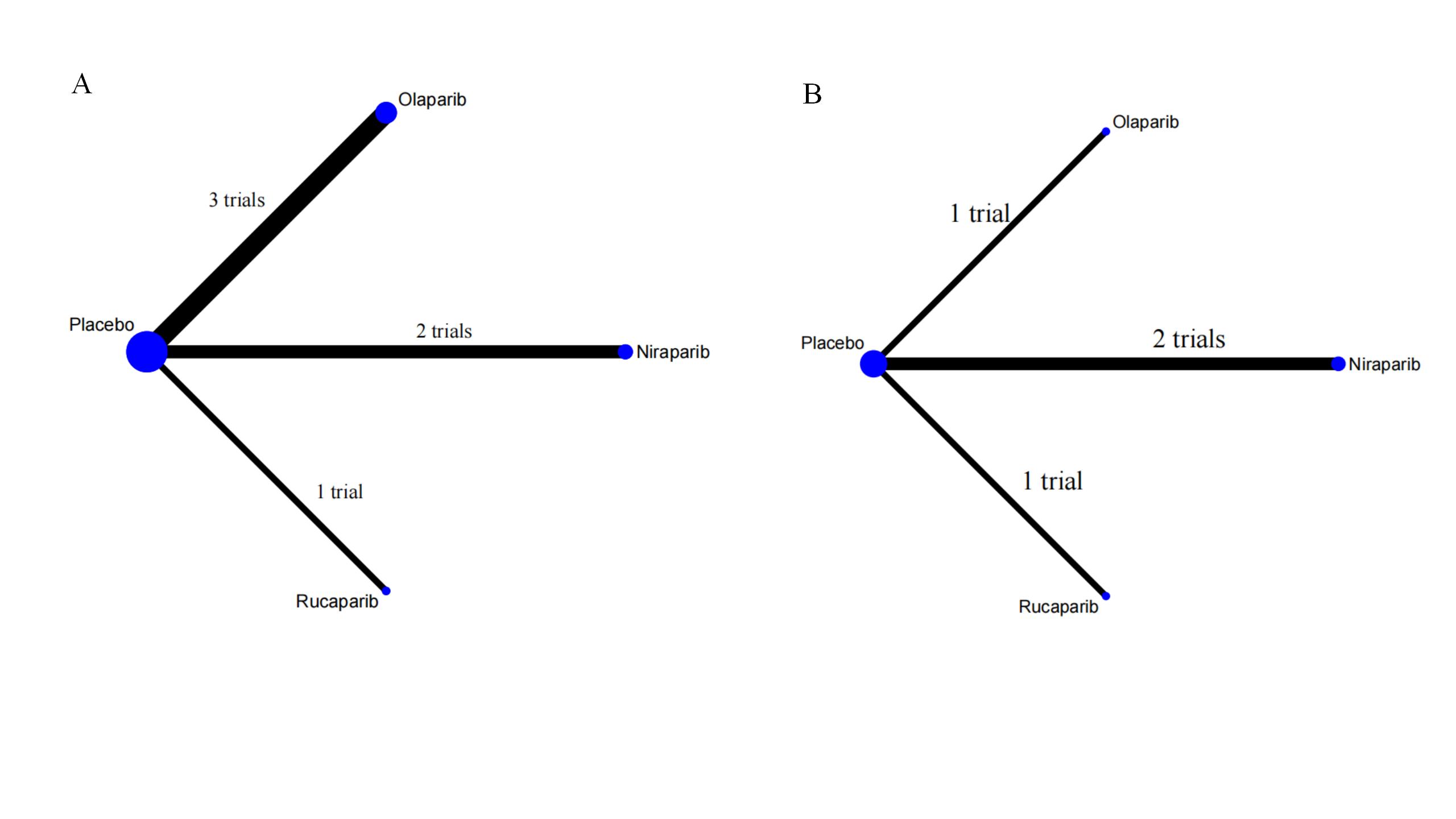

Supplement: Supplementary Figure 3 — Network plot of comparisons on progression-free survival of treatments in subgroups of patients with ovarian cancer. (A) Comparisons on progression-fee survival in BRCA mutation population. (B) Comparisons on progression-fee survival in wild-type BRCA population. [file Image_3.jpeg]

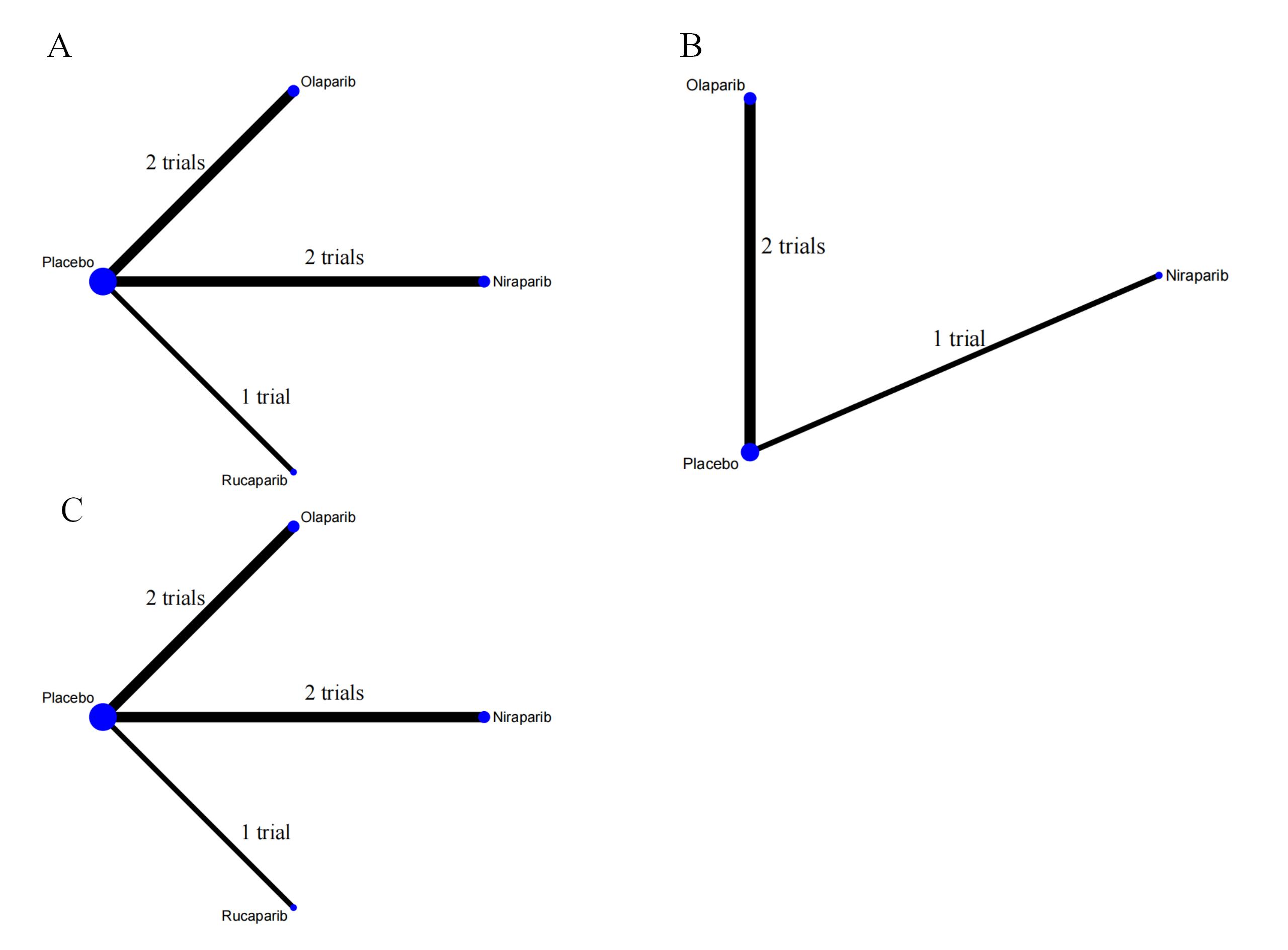

Supplement: Supplementary Figure 4 — Network plot of different outcomes in patients with ovarian cancer for sensitivity analysis. (A) Comparisons on progression-free survival in patients with ovarian cancer. (B) Comparisons on overall survival in patients with ovarian cancer. (C) Comparisons on grade ≥3 adverse events in patients with ovarian cancer. [file Image_4.jpeg]

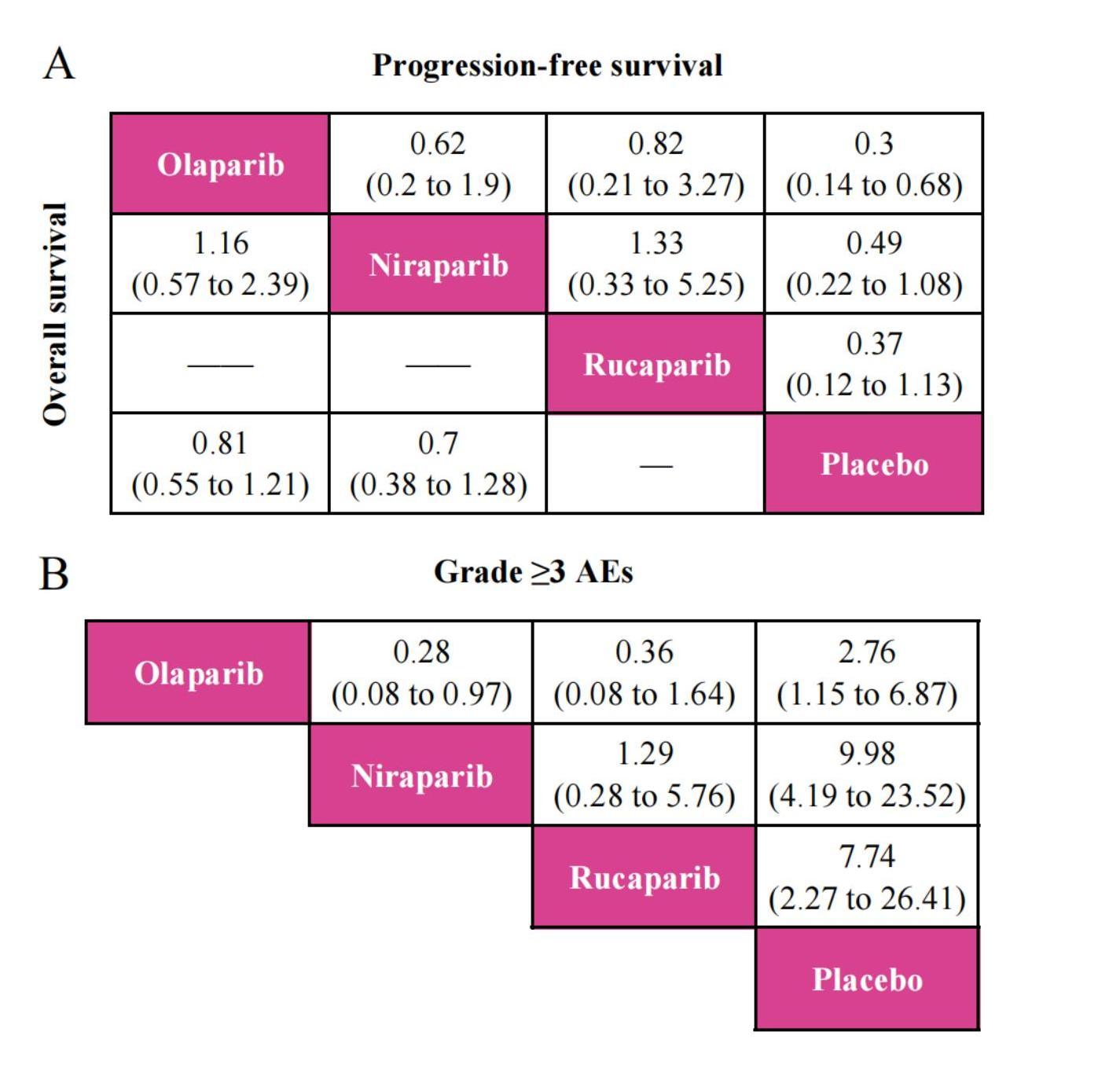

Supplement: Supplementary Figure 5 — Pooled estimates of the sensitivity analysis. (A) Pooled hazard ratios (95% confidence intervals) for progression-free survival (upper triangle) and overall survival (lower triangle). (B) Pooled odds ratios (95% confidence intervals) for grade≥3 adverse events. [file Image_5.jpeg]
